# Honest signals and sexual conflict: Female lizards carry undesirable indicators of quality

**DOI:** 10.1002/ece3.7598

**Published:** 2021-05-02

**Authors:** Braulio A. Assis, Julian D. Avery, Catherine Tylan, Heather I. Engler, Ryan L. Earley, Tracy Langkilde

**Affiliations:** ^1^ Department of Biology The Pennsylvania State University University Park PA USA; ^2^ Intercollege Graduate Degree Program in Ecology The Pennsylvania State University University Park PA USA; ^3^ The Department of Ecosystem Science and Management The Pennsylvania State University University Park PA USA; ^4^ Department of Biological Sciences University of Alabama Tuscaloosa AL USA

**Keywords:** color, condition dependence, female ornamentation, honesty mechanism, intralocus sexual conflict, *Sceloporus*, sexual antagonism

## Abstract

Sex differences in animal coloration often result from sex‐dependent regulatory mechanisms. Still, some species exhibit incomplete sexual dimorphism as females carry a rudimentary version of a costly male trait, leading to intralocus sexual conflict. The underlying physiology and condition dependence of these traits can inform why such conflicts remain unresolved. In eastern fence lizards (*Sceloporus undulatus*), blue iridophore badges are found in males and females, but melanin pigmentation underneath and surrounding badges is male‐exclusive. We track color saturation and area of badges across sexual maturity, and their relationship to individual quality (body condition and immunocompetence) and relevant hormones (testosterone and corticosterone). Saturation and testosterone were positively correlated in both sexes, but hormone and trait had little overlap between males and females. Saturation was correlated with body condition and immunocompetence in males but not in females. Co‐regulation by androgens may have released females from resource allocation costs of color saturation, even when in high condition. Badge area was independent of testosterone, but associated with low corticosterone in females, indicating that a nonsex hormone underlies incomplete sexual dimorphism. Given the evidence in this species for female reproductive costs associated with ornamentation, this sex‐nonspecific regulation of an honest signal may underlie intralocus sexual conflict.

## Graphical abstract

Sex‐specific regulatory mechanisms may allow adaptive sexual dimorphism despite genetic similarities between sexes. However, many species exhibit sexually selected ornaments with incomplete sexual dimorphism, even though costly to one sex. We show that colorful badges of eastern fence lizards are condition dependent and honest signals of quality in males, but not in females, and that partial regulation of this trait by a sex‐nonspecific hormone (corticosterone) is one factor preventing complete sexual dimorphism and the resolution of intralocus sexual conflict.

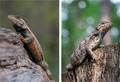

## 1 INTRODUCTION

Animals of many species advertise their quality as competitors and mates using a wide array of behavioral displays and elaborate ornaments (Johnstone, 1996). In many species, stronger competition among males for access to mating opportunities favors the development of colorful ornaments in males exclusively (Andersson, 1994; Emlen & Oring, 1977). In females, adaptive benefits of ornament expression may be reduced when opportunities to mate are not limited and the benefits of advertising condition do not offset the costs of producing and carrying conspicuous signaling traits—particularly as investment in fecundity can be compromised in the process (Fitzpatrick et al., 1995; Nordeide et al., 2006; but see Chenoweth et al., 2006). For this reason, ornaments are typically absent in females (Hamilton & Zuk, 1982; Price & Birch, 1996). In some species, however, condition‐dependent signals in females can still be under directional selection in some social contexts (Cornwallis & Birkhead, 2007; Griggio et al., 2009; Hegyi et al., 2008) and therefore be adaptive regardless of their selection in males (Amundsen, 2000; Karubian, 2013; Price & Birch, 1996). Importantly, ornamentation in females does not always follow a binary pattern of expression across species; in some, females carry an ornament that is rudimentary compared to those of males (Kraaijeveld et al., 2007). Whether these rudimentary traits in females still convey relevant information in social interactions (LeBas, 2006; LeBas et al., 2003; Rubenstein & Lovette, 2009) or are merely genetically linked to a trait that is strongly selected for in males (Lande, 1980) seems to be inconsistent across systems (Kraaijeveld et al., 2007). If rudimentary signals are not advantageous to females, their expression can be predicted to reduce female fitness in a scenario of intralocus sexual conflict (Arnqvist & Rowe, 2005; Bonduriansky & Chenoweth, 2009; Chapman et al., 2003; Pennell & Morrow, 2013). Understanding the physiological factors underlying ornament expression between sexes, as well as the information content associated with these traits may help clarify what prevented some species from evolving toward complete sexual dimorphism.

For condition signals to be informative and reliable (“honest”), theory predicts that message and signal must be physiologically linked (Biernaskie et al., 2014; Maynard Smith & Harper, 1995), or that development of the signaling trait must be directly dependent upon an individual's acquired resources (Zahavi, 1975). Instances where females bear a less elaborate version of a trait that is presumably condition‐dependent in males could indicate that: (a) female condition is poorer than that of males, (b) the trait is not an honest signal, as females can express reduced ornaments but be of high condition, or (c) the honesty mechanism is expressed only in males due to genetic or epigenetic factors underlying the trait (Adkins‐Regan, 1998; Cox et al., 2017; Kimball & Ligon, 1999; Wright et al., 2018, 2019). Sex‐specific hormone concentrations are well established as important drivers of sexual dimorphism (Ketterson et al., 2005; Owens & Short, 1995; Staub & De Beer, 1997; but see Goymann & Wingfield, 2014), and a number of laboratory experiments have been successful in using androgen supplementation to induce the development of male‐specific traits in females of many species (Cox et al., 2005; Hayes & Menendez, 1999; Lindsay et al., 2016; Nespor et al., 1996; Peters, 2007; Pollock et al., 2017). This suggests that in some species both sexes share downstream regulatory networks responsible for the expression of sexually dimorphic traits, but these require sex‐specific hormone levels found only in the sex in which the trait improves fitness. Therefore, co‐regulation of signaling traits by condition dependence and sex‐nonspecific hormones may be one mechanism underlying incomplete sexual dimorphism of honest signals. Species carrying conspicuous, multicomponent traits with incomplete dimorphism between sexes should be excellent systems for investigating the physiological underpinnings that maintain incomplete sexual dimorphism and, potentially, intralocus sexual conflict.

**FIGURE 1:**
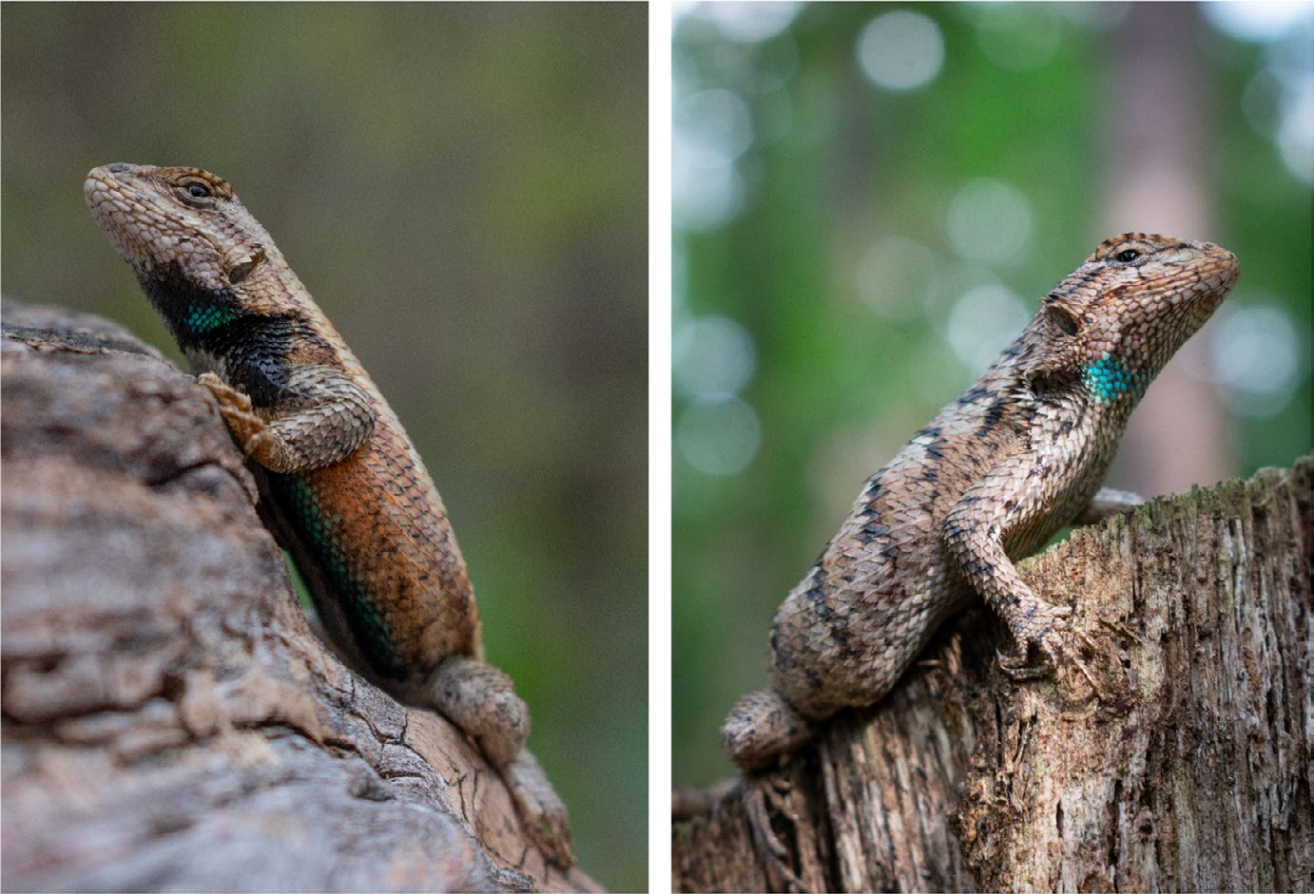
Left: male eastern fence lizard exhibiting blue iridophore badge on throat, with surrounding and underlying (not visible) melanin; right: female eastern fence lizard with well‐developed throat badge, although lacking any visible melanin

Many lizard species in the Phrynosomatidae family exhibit conspicuous, sexually selected ornaments (Wiens, 1999). In eastern fence lizards (*Sceloporus undulatus*), males display vivid blue badges surrounded by a black border in the abdominal and throat regions (Figure 1, left). Electron microscopy determined that badges consist of a layer of iridophores containing guanine platelets reflecting mostly blue wavelengths (i.e., structural color) above a layer of melanophores containing melanin (Morrison et al., 1995). Melanin underlying the iridophores is assumed to absorb incoherently scattered light, allowing badges to display a purer, more saturated blue signal (Maia et al., 2009). The black border surrounding badges in male fence lizards seems to result from the melanophore layer extending beyond the limits of iridophores, potentially increasing the contrast and conspicuousness of blue badges to receivers (Bókony et al., 2003). In females, color is often entirely absent, but some individuals develop blue patches considerably fainter than those of males ( Figure 1, right; Assis et al., 2020; Swierk & Langkilde, 2013). Even among these partially ornamented females, the black melanin border is extremely rare, and the iridophore portion of the badge exhibits fainter “turquoise” hues that suggest the underlying melanophore layer may be absent altogether (Quinn & Hews, 2003; Shawkey & Hill, 2006). Interestingly, female supplementation with androgens leads to the development of badges comparable to male badges in terms of saturation of the blue component and presence of surrounding black (Cox et al., 2005; Pollock et al., 2017), both of which may be attributed to an increase in melanin density beneath and around iridophores (Quinn & Hews, 2003). Therefore, it appears that although some females develop iridophores, melanophores may be androgen‐dependent (Bókony et al., 2008) and the true sex‐specific ornament component in fence lizards.

In male *S*. *undulatus*, badge quality (saturation or size) seems to be important in female mate choice (Swierk et al., 2012) and is correlated with body size (Langkilde & Boronow, 2010). Males may use more developed ornaments to signal elevated androgen levels and, indirectly, the ability to secure and defend resources, as suggested in other systems (Bókony et al., 2008; Jawor & Breitwisch, 2003). However, an association between badge quality and condition traits—such as energy reserves and immune response (Pérez‐Rodríguez et al., 2006)—has not been clearly demonstrated in this species (Langkilde & Boronow, 2012; Pollock et al., 2017). Among female *S*. *undulatus*, a signaling function of rudimentary badges is even less clear, as few fitness benefits have been associated with the presence of badges (Assis et al., 2018). In fact, females bearing residual ornaments appear to incur costs: laboratory mate choice trials indicate that males were more likely to court unornamented females, and ornamented females had lower reproductive output (Swierk & Langkilde, 2013). The lack of preference for ornamented females could be a product of sex misidentification. During laboratory manipulations, female fence lizards painted with artificial badges were treated aggressively by other males, who exhibited agonistic displays typical of territorial contests (Cooper & Burns, 1987). With the lack of obvious direct fitness benefits of ornamentation in female fence lizards, and the polymorphic nature of the trait in females, it is not clear which selective pressures might permit the coexistence of the two phenotypes. The proportion of ornamented females varies significantly across populations, ranging from 40% to 90% of females surveyed (T.L. unpublished data), which warrants further investigation of the factors underlying this trait.

We explored the eastern fence lizard system to investigate the evolution and maintenance of ornaments with incomplete sexual dimorphism, their condition dependence, and their potential for intralocus sexual conflict. We first tested the hypothesis that color saturation and area of throat badges have sex‐specific relationships with proxies of body condition (Megía‐Palma et al., 2016) and immune response (Dufva & Allander, 1995; Roberts et al., 2004). Next, we hypothesize that saturation and badge area are correlated with sex and nonsex hormones as a potential cause of incomplete sexual dimorphism for this trait. We investigate the stress‐relevant hormone corticosterone (CORT) as a candidate for this hypothesis due to its relationship with color development and immune response in other systems (French et al., 2007; San‐Jose & Fitze, 2013; but see MacLeod et al., 2019). We evaluate these relationships both from the perspective of unbiased coloration scores and of coloration as perceived by an iguanid visual sensitivity model. Support for our hypotheses would indicate that (a) badges signal quality in fence lizards and that the dependence of color saturation on male‐specific androgen titers allowed females to partially eliminate the physiological and social costs of ornament maintenance; and (b) the condition dependence of badge area in both males and females and the lack of a sex‐specific regulatory mechanism for this trait component underlie a scenario of intralocus sexual conflict: high‐condition females develop condition‐dependent traits that carry fitness costs.

## 2 METHODS

### 2.1 Study organism

Eastern fence lizards (*Sceloporus undulatus*) were raised in the laboratory from eggs obtained from gravid females collected at field sites in Tennessee (Land Between the Lakes National Recreation Area (LBL) and Edgar Evins State Park (EE)) and Arkansas (St. Francis National Forest and private lands in Lee County (SF)). Initial sample sizes for each population were as follows: LBL: 10 females and seven males across two clutches; EE: five females and seven males from one clutch; SF: 47 females and 44 males across nine clutches. Husbandry protocols can be found in the Supporting Information.

Juveniles were monitored for 11 months, during which we quantified badge saturation and area (see Color quantification) and collected blood samples for hormone analyses (see Hormone quantification) at three time points in relation to an individual's hatch date: age group 1 (189 ± 4 days), age group 2 (253 ± 4 days), and age group 3 (323 ± 2 days). At age group 3, all individuals were at, or close to, maturity (snout‐to‐vent length >54 mm, Cooper & Vitt, 1989). At this end point, number of surviving offspring according to maternal site of origin were as follows: LBL: six females and three males across two clutches; EE: two females and four males from one clutch; SF: 15 females and 12 males across seven clutches. We estimated body condition of individuals at age group 3 by measuring and extracting the residuals of a linear regression of log(body mass) on log(snout‐to‐vent length). Immune response was measured for individuals at 274 ± 2 days of age, closest to age group 2 (see Hormone and immune response assays).

### 2.2 Color quantification

We quantified badge reflectance using an Ocean Optics Jaz UV/VIS spectrometer and the R package *pavo* (Maia et al., 2019) with minor modifications that better account for unornamented females (Assis et al., 2020). To estimate color conspicuousness as perceived by conspecifics, we fit the color data to a visual sensitivity model using visual parameters from the most closely related species for which these data are available, the crotaphytid lizard *Crotaphytus dickersonae* (Macedonia et al., 2009). Our method for calculating saturation in these temperature‐dependent badges (Langkilde & Boronow, 2012; Stephenson et al., 2017) and information on the *Crotaphytus* visual model is detailed in previously published work (Assis et al., 2020) and in the Supporting Information.

Although male *S*. *undulatus* exhibit pairs of badges on their throats and abdomens, in females, badges are almost always seen only on their throats (unpublished data). To facilitate comparisons between sexes, we only quantified ornamentation on the lizards’ throats. To quantify badge area, we used ventral photographs of males and females and measured the area of the blue portion of the throat badges using ImageJ (Schneider et al., 2012). Because female badges typically do not exhibit melanized scales on the badge margin as males do, we measured only the blue portion of badges, also to facilitate comparison across sexes. Additional details can be found in the Supporting Information.

### 2.3 Hormone and immune response assays

We collected blood from the lizards’ postorbital sinus to quantify testosterone (T) and corticosterone (CORT) levels at the three age groups. Plasma steroid extractions and assay procedures are detailed in the Supporting Information. Aliquots from a single pool of hormone were run at the start and end of each plate for intra‐ and interassay control (intraassay: all CV <11.5%; interassay: all CV <14.1%).

We used the phytohemagglutinin (PHA) skin test to measure the cell‐mediated immune response of lizards approximately three weeks after the second color measurement (at 274 ± 2 *SD* days of age). Increased tissue swelling following injection of the PHA lectin used here (PHA‐L) is indicative of an increased T lymphocyte response (Tylan & Langkilde, 2017). The immune response protocol can be found in the Supporting Information.

### 2.4 Statistical analyses

For all analyses, we used R v. 4.0.3 (RCoreTeam, 2020) and the packages *lme4* 1.1–25 (Bates et al., 2015) and *lmerTest* 3.1‐3 (Kuznetsova et al., 2019) for linear mixed‐effects models. We first used two‐way ANOVA’s and Tukey's HSD to investigate how sex and age class relate to badge saturation, badge area, and plasma T. Next, we assessed the relationship between the two badge components (saturation and area) for males and females at age group 3 (our final measuring point, at maturity) using a linear mixed‐effects model. This model contained saturation as the response variable and individual sex and badge area as predictors, along with a sex‐by‐badge‐area interaction term. For this and all subsequent models, maternal identity was nested within maternal population of origin (of the three populations; see Animal collection) as a random effect.

Our next objective was to test the relationships between hormones, proxies for individual quality (body condition and immune response), and degree of ornamentation. We built three models to investigate each component of the ornament: raw saturation (model 1), saturation corrected by the iguanid visual model (model 2), and badge area (model 3). This was done for badge data collected at age group 3 because lizards had reached maturity and had developed adult badges. Predictors for the three models were identical: body condition, T, CORT, and individual sex. To identify possible sex‐dependent effects, we also included two‐way interaction terms of sex by body condition, by T, and by CORT. Androgens and glucocorticoids (such as T and CORT) can exhibit an interactive effect on sexually selected traits (Puts et al., 2016), and for this reason, we also included an interaction term for T by CORT.

Because immune response trials were carried out when individuals were younger, we used three separate models to compare immune response to badge development at the closest matching age (age group 2). Models 4, 5, and 6 (for raw saturation, corrected saturation, and badge area at age group 2, respectively) were fitted using the same predictors as models 1–3, with the exception that body condition was substituted by immune response.

Testosterone exhibits a nonsynchronous relationship with badge development in fence lizards, with color intensity correlating to androgen levels measured at younger ages (Cox et al., 2005; Pollock et al., 2017). This is likely due to the phenotype being organized by hormone levels during a prior developmentally sensitive window (Dean et al., 2012). For this reason, in each model described above, we included T levels from one age group prior to age group of badge values. That is, models 1, 2, and 3 tested for the effects of T levels at age group 2 on badge traits at age group 3; models 4, 5, and 6 tested for the effects of T at age group 1 on badge traits at age group 2. We contemplated following a similar approach with the other predictors to test for the effect of past body condition, immune response, and CORT on organizing future badge development. However, the goal for this study was to understand the potential of this trait to signal condition to conspecifics, and we concluded that any relationships between future signal strength and past condition would not characterize an informative signal. Similarly, we predicted that CORT would be more responsive to social interactions potentially mediated by ornamentation, given our animal housing design (Yang & Wilczynski, 2003). Thus, we tested the relationships between body condition, immune response, and CORT against synchronous values of badge saturation and area.

We performed model selection via stepwise removal of nonsignificant interaction terms and main effects. Candidate models were compared via AICc using the *MuMIn* 1.43.17 package (Bartoń, 2018), and the ones with lowest scores (at least two AICc units below) were considered final. Model diagnostics were checked by visual inspection of residual versus fitted values, and we looked for highly influential data points (Cook's distance >1). At age group 2, a highly influential data point was detected for T (6.8 times the standard deviation of the sample), and this individual was removed from models 1 and 2 in which T was a predictor. However, since model selection eventually removed T as a predictor from model 3 (see Results), we reintroduced this individual in the final model. Data distribution for T at age groups 1 and 2 showed significant skew and we considered log‐transforming these predictors to improve model fit. However, doing so resulted in models with a reduced overall fit (more skewed residuals versus fitted values) so we chose to keep T values untransformed. All final models were checked for multicollinearity via variance inflation factor (VIF) using the *car* 3.0‐10 package (Fox & Weisberg, 2019). In model 6, the covariate Sex had a very high VIF (23.3), but due to the critical role of sex differences in our predictions, this variable was maintained. No predictors in other models showed a VIF >10.

Lastly, to determine whether saturation differences between individuals might be perceptible to a lizard observer (and potentially function as a distinguishable signal), we calculated chromatic “just noticeable differences” (JNDs) using *pavo's* function *coldist* with our visual sensitivity model parameters. The appropriate Weber fraction (receptor noise coefficient) for this species has not yet been determined, although studies in amphibians and other *Sceloporus* lizards have employed a Weber fraction of 0.05 (Robinson & Gifford, 2018; Siddiqi et al., 2004). Chromatic JND results using a Weber fraction of 0.05 were qualitatively identical to the more frequently used and more conservative Weber fraction of 0.1 for chromatic vision (Olsson et al., 2018; Vorobyev & Osorio, 1998), and thus, we present results obtained with the latter (see Results). We calculated color JNDs for each sex separately, and within each sex, between the individual with the highest saturation (*x*_1_) against the individual with the second highest saturation (*x*_2_), and between *x*_1_ and the individual with the lowest saturation (*x*_n_). A JND ≥1 indicates that colors are distinguishable to an observer of the closely related lizard species, *C*. *dickersonae*.

## 3 RESULTS

**FIGURE 2:**
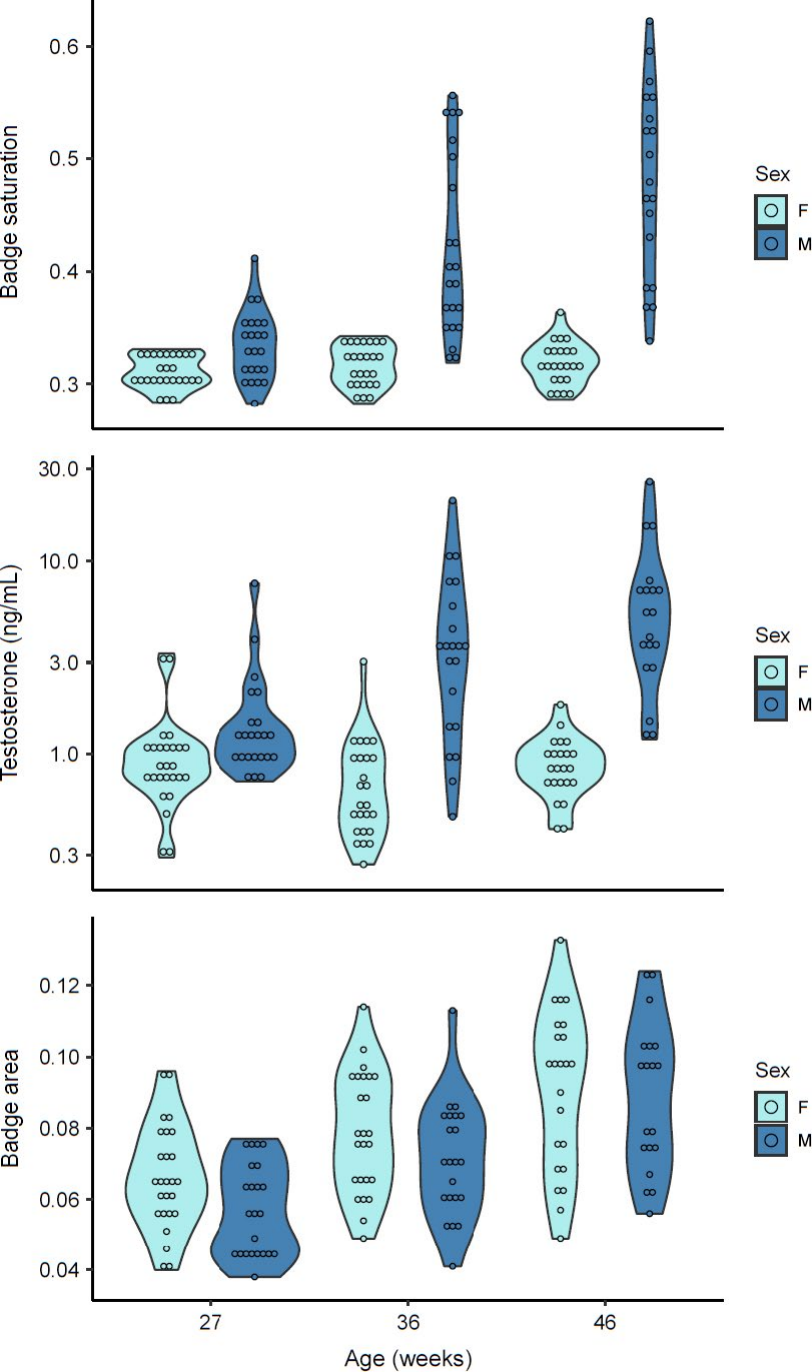
Violin plot showing variation in badge saturation, testosterone levels, and absolute throat badge area between males and females at three age classes: 27 weeks (females, *n* = 26, males, *n* = 23); 36 weeks (females, *n* = 25, males, *n* = 22); and 46 weeks of age (females, *n* = 23, males, *n* = 19). Widths of violins represent density of individuals at different levels of badge saturation, testosterone, and badge area

The saturation of throat badges steadily increased up to maturity in male lizards (ANOVA, age group by sex: *F*_5,132_ = 45.08, *p* < .001; Tukey's HSD for males between age groups 1 and 2 and between age groups 2 and 3: *p* < .01) but remained relatively constant in females (Tukey's HSD for females between age groups 1 and 3: *p* = .992; Figure 2). A similar pattern was observed for T levels (ANOVA, age group by sex: *F*_5,132_ = 5.032, *p* < .001; Tukey's HSD for males between age groups 1 and 2: *p* = .025; between age groups 2 and 3: *p* = .98; females between age groups 1 and 3: *p* = .999; Figure 2). However, badge areas increased with age similarly for both sexes (ANOVA, age group by sex: *F*_5,132_ = 13.22, *p* < .001; Tukey's HSD for males and females between age groups 1 and 3: *p* < .001) and were not significantly different between males and females in age group 3 (*p* = .998). All patterns above are shown in Figure 2.

At maturity (age group 3), the relative size of the blue badge was unrelated to its saturation (*t* = 0.392, *df* = 37.994, *p* = .697), for both males and females (badge area by sex interaction: *t* = −1.64, *df* = 36.094, *p* = .108).

**FIGURE 3:**
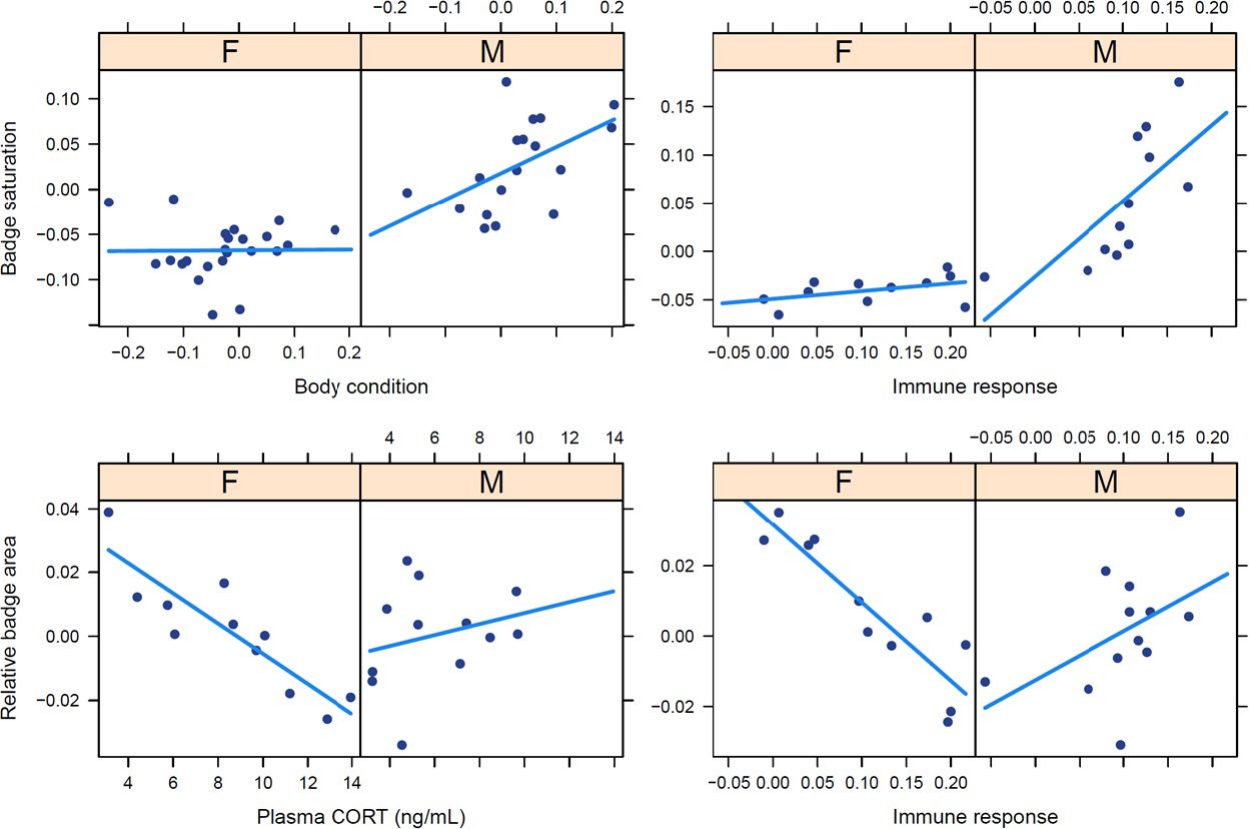
Sex interaction effects on badge saturation (corrected for body temperature at measurement) for body condition (residuals from linear regression of log[body mass] on log[snout‐to‐vent length]; top left) and immune response (tissue swelling after PHA assay; top right), and on relative throat badge area for plasma corticosterone concentrations (bottom left) and immune response (bottom right). F, females; M, males

**FIGURE 4:**
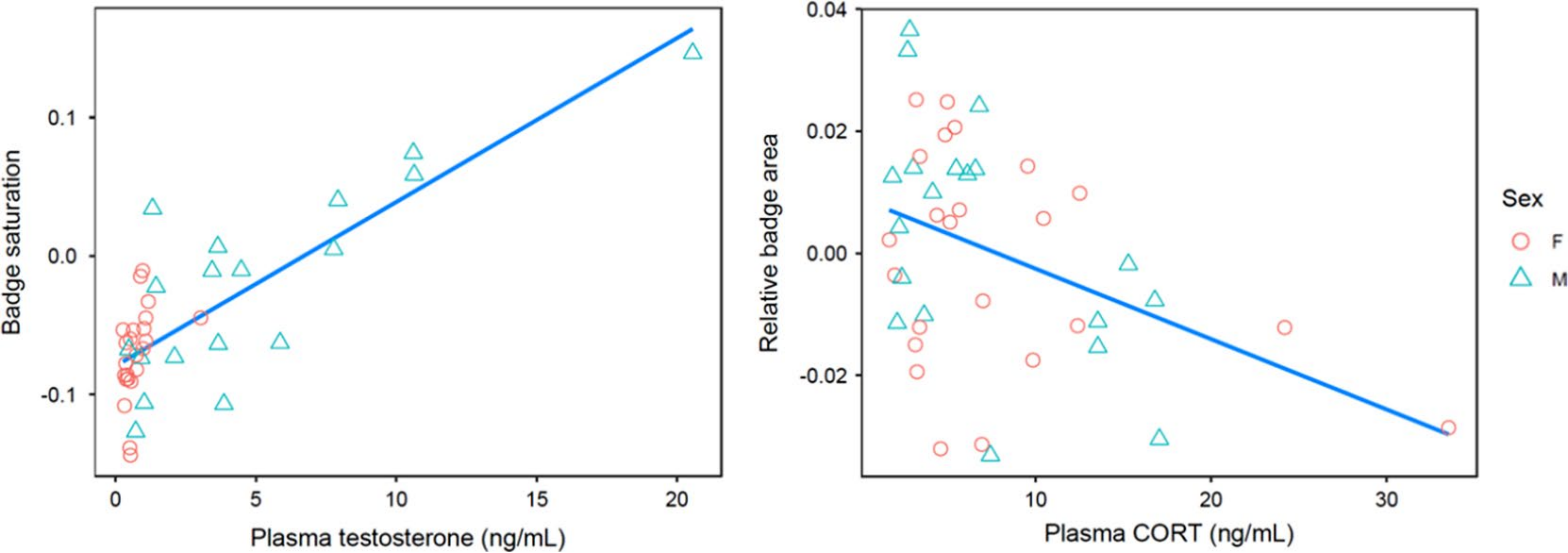
Relationship between plasma testosterone and throat badge saturation and between plasma corticosterone and relative throat badge area, for females (circles) and males (triangles), at maturity

All following results are outputs from the most parsimonious version of each of six full models, based on AICc (Bartoń, 2018). In model 1 (raw saturation in age group 3—maturity), the interaction between body condition and sex showed a strong, significant relationship with raw badge saturation, where males with greater body condition carried more saturated badges but females did not (Table 1, model 1; Figure 3). T levels measured in age group 2 also showed a significant positive relationship with raw badge saturation at age group 3 (Table 1, model 1; Figure 4); note that the T by sex interaction was not significant, indicating that this relationship was consistent between sexes. In model 2 (saturation corrected by visual model, at maturity), however, body condition was no longer a significant predictor of saturation after it was corrected for the *C*. *dickersonae* visual model (Table 1, model 2). In model 3 (relative badge area at maturity), a very strong interactive effect between sexes was detected for immune response, with a positive relationship for males and a neutral one for females (Table 1, model 3; Figure 3), as well as a negative association between badge area and CORT (Table 1, model 3; Figure 4), which did not vary between the sexes (the interaction term with sex was not significant). Of note, there was no significant relationship between T and badge area (Table 1, model 3).

**TABLE 1:** The most parsimonious models showing the effects of body condition, sex, and hormone levels on: model (1) badge saturation (reflectance on the range of peak reflectance ±50 nm divided by total reflectance between 300 and 700 nm, corrected for individual body temperature during measurement) at maturity (46 weeks of age); model (2) badge saturation corrected for a lizard visual sensitivity model (*Crotaphytus dickersonae*), at maturity; model (3) badge area relative to head area, at maturity; and of immune response, sex, and hormone levels on: model (4) badge saturation prior to maturity (36 weeks of age); model (5) badge saturation corrected for the visual sensitivity model, prior to maturity; model (6) relative badge area, prior to maturity. β: slope coefficient; *SE*: standard error. Predictors and p‐values in bold are statistically significant at α = 0.05. For all models, maternal identity nested within site of origin was included as a random effect

			β	*SE*	*t*	*p*
Maturity	Saturation	Model 1				
		Intercept	−0.084	0.011	−7.725	<.001
		**Body condition: Sex**	0.288	0.141	2.042	.**049**
		**T**	0.012	0.002	6.534	**<.001**
		CORT	0.001	0.001	0.872	.389
		Body condition	0.004	0.099	0.040	.968
		Sex	0.085	0.015	5.761	<.001
	Visual model	Model 2				
		Intercept	−0.149	0.019	−7.720	<.001
		**T**	0.023	0.004	5.295	**<.001**
		**Sex**	0.180	0.034	5.285	**<.001**
	Area	Model 3				
		Intercept	0.009	0.005	1.640	.121
		Body condition	−0.068	0.035	−1.945	.059
		**CORT**	−0.001	0.000	−2.394	.**022**
Prematurity	Saturation	Model 4				
		Intercept	−0.048	0.055	−0.870	.397
		**Immune response: Sex**	0.704	0.273	2.575	.**020**
		**T: Sex**	0.063	0.021	2.935	**<.01**
		Immune response	0.080	0.197	0.407	.689
		T	−0.011	0.017	−0.649	.526
		Sex	−0.040	0.044	−0.904	.379
		CORT	0.001	0.004	0.354	.728
	Visual model	Model 5				
		Intercept	−0.120	0.033	−3.645	.056
		**Sex**	0.229	0.030	7.602	**<.001**
	Area	Model 6				
		Intercept	0.063	0.025	2.517	.023
		**Immune response: Sex**	0.361	0.113	3.208	**<.01**
		**CORT: Sex**	0.006	0.003	2.351	.**043**
		Immune response	−0.222	0.082	−2.699	.016
		CORT	−0.005	0.002	−2.432	.027
		Sex	−0.090	0.029	−3.091	.007
		T	0.002	0.004	0.651	.525

In model 4 (age group 2—prematurity), raw badge saturation was positively associated with immune response and T in males, but not in females (immune response: sex interaction; Table 1, model 4, Figure 3; T: sex interaction, Table 1, model 4). No main effects were significant. However, when saturation was corrected for the *Crotaphytus* visual model, only sex retained statistical significance (Table 1, model 5). Regarding relative badge area (age group 2, Table 1, model 6), interactions of sex with both immune response and CORT showed significant relationships: stronger immune responses were associated with smaller badges in females, but larger badges in males (Figure 3); CORT was negatively associated with badge size in females, but showed a weak positive relationship in males (Figure 3).

Chromatic JND analyses indicate that color differences between males at age group 3 are distinguishable to a lizard observer (even differences between the two most saturated males). Among females, differences between the highest‐ and lowest‐ranked individuals were distinguishable, but those between the two highest saturated females were not (Table 2).

**TABLE 2:** Chromatic “just noticeable differences” (JNDs) based on the *Crotaphytus dickersonae* visual sensitivity model and Weber fraction of 0.1, for females and males. Comparisons are between individuals with *x*_1_: highest saturation; *x*_2_: second highest saturation; *x*_n_: lowest saturation. Colors with JNDs >1 are considered distinguishable to the observer

	*x*_2_	*x*_n_
Females		
*x*_1_	0.152	1.245
Males		
*x*_1_	2.256	5.928

## 4 DISCUSSION

Traits that signal condition can be sexually dimorphic if they are co‐regulated by sex‐specific physiology (Owens & Short, 1995). However, signaling traits with multiple components may exhibit a combination of plasticity and hormonal regulation (Cox et al., 2016; Ketterson et al., 2009), which can lead to incomplete sexual dimorphism. We show that upon reaching maturity, female fence lizards can develop throat badges that are equivalent in relative size but significantly less saturated in color than those of males. Color saturation was higher in males with greater body mass relative to body length and in males with a greater swelling response following an immune challenge. However, neither pattern was seen in females. More saturated badges were associated with high testosterone levels in both sexes, but the ranges for saturation and testosterone had little overlap across sexes. This suggests that color saturation responded to an individual's quality only in males due to its regulation by androgens. Sexually monomorphic badge area was independent of testosterone, but negatively associated with CORT in females. It is important to note that our results are correlative, and we exercise caution on inferring causation in the patterns observed here. Still, the sex‐interactive directionality of the proxies for condition and hormones on ornamentation support our hypotheses and predictions, and our interpretations of these patterns suggest important avenues for future investigation. Our findings indicate how a lack of complete regulation of a sexually selected trait by sex hormones may prevent the resolution of intralocus sexual conflict, as a condition‐dependent trait correlates with a nonsex hormone and is expressed even when costly to one sex (Cooper & Burns, 1987; Swierk & Langkilde, 2013).

In our study system, the structural blue/green component of badge color (iridophore area) was associated with immunocompetence and indicators of physiological stress (CORT). Such links between CORT and structural coloration have also been seen in eastern bluebirds, irrespective of their sex (Grindstaff et al., 2012). This illustrates how trait condition dependence can develop in both sexes, even when the signaling potential in females is unclear. In birds, the occurrence of sexual dichromatism not only varies considerably across species, but also with respect to the types of color‐producing mechanisms—structural, melanin, or carotenoids (Owens & Hartley, 1998). Similarly, sexual dimorphism in *S*. *undulatus* seems to vary with the nature of its color components: the size of the structural blue portion of badges was associated with CORT; color saturation—likely driven by underlying melanin pigmentation—was associated with testosterone.

Although we detected significant associations between badges and proxies for quality in males, the models for color saturation corrected for the iguanid visual model showed no such relationships, contrary to our predictions. Still, our analyses of uncalibrated saturation showed that these quality traits are associated with ornament development independent of the visual sensitivity of signal receivers. Interspecific competition for resources is common among lizards (Dunham, 1980; Langkilde & Shine, 2004; Smith, 1981), and it is possible that competing species sympatric with *S*. *undulatus* may possess the visual acuity to detect these relationships (Leal, 1999; Leal & Rodríguez‐Robles, 1995). Determining the visual sensitivity parameters for *S*. *undulatus* and other species would allow us to explore the signaling potential of this ornament both intra‐ and interspecifically. Furthermore, our analyses for chromatic “just noticeable differences” (JND) suggest that, among males, this signal is easily distinguishable, but among females it is distinguishable only for the most divergent levels of saturation (i.e., only between the highest‐ and lowest‐ranked females). This corroborates the idea that *S*. *undulatus* badges in males can function as a signal to competitors and mates, but the rudimentary color in females may have limited signaling function.

Despite being widespread among phrynosomatids, the message content of colorful badges in males had so far been ambiguous (Goodlett & Stephenson, 2019; Langkilde & Boronow, 2010; Robinson & Gifford, 2019). To our knowledge, no study in this family had addressed correlates of condition in both males and females in relation to the two components of this trait (saturation and area) separately. Testosterone is a known driver of aggressive and territorial behaviors in fence lizards (Moore, 1988) and potentially increased resource acquisition (but see Klukowski et al., 2001), leading to higher body condition; however, accounting for T in our analyses allowed us to disentangle potential confounding effects of body condition and T (and associated aggressiveness). Nevertheless, the mechanism that maintains signal honesty in this species remains unclear and conforms to both the index hypothesis (Biernaskie et al., 2014; Maynard Smith & Harper, 1995) and the costly signal hypothesis (Zahavi, 1975): males in greater condition may have more resources to allocate into melanin synthesis; and melanophore and iridophore development may be physiologically tied to immune response via androgens and stress hormones, respectively. Importantly, these frameworks are not mutually exclusive (Weaver et al., 2017), and both mechanisms may work in conjunction to maintain an informative signal in male fence lizards. Understanding the fitness implications of these patterns under natural field conditions would be valuable.

Although less pronounced than in males, badges in females carry reproductive costs (Cooper & Burns, 1987; Swierk & Langkilde, 2013). Indirect benefits related to badge presence such as increased locomotor performance, however, may allow female ornamentation to persist as a polymorphic trait through increased survival (Assis et al., 2018). Still, other scenarios and life stages where female ornamentation could be advantageous remain unexplored. In the juvenile stage when male and female *S*. *undulatus* badges are equivalent in size and saturation, it is possible that quality signals in females may be advantageous in defending resources from conspecifics (Ruby & Baird, 1993). Social competition experiments among juvenile males and females could give us new insight into the significance of female ornamentation in this and other species with sexually monomorphic ornaments prior to maturity. At maturity, when male ornaments become dramatically more pronounced, however, the signaling potential in female badges may depreciate. Importantly, the association between CORT and badge area in females may lead to fitness costs. In a different study, females with elevated CORT produced eggs with significantly less protein, and behavioral trials with their offspring showed that they spent more time in hiding versus exploring their surroundings (Ensminger et al., 2018). If males show a preference for less ornamented females (Swierk & Langkilde, 2013), and less ornamented females have higher CORT levels (this study), then ornaments may be costly to females in mating settings due to male harassment and mating avoidance ​(Cooper & Burns, 1987; Swierk & Langkilde, 2013). Conversely, more ornamented, low CORT females may be treated aggressively and avoided as mates but may produce eggs with higher protein (Ensminger et al., 2018). Female reproductive costs associated with ornamentation suggest that female *S*. *undulatus* carry a trait that may be maladaptive in some contexts and characterize sexual conflict (Chapman, 2006).

Across the *Sceloporus* phylogeny, multiple losses and gains of conspicuous color in males and females have occurred (Wiens, 1999), and besides the likely role for natural selection, intralocus sexual conflict or female preference for male signals may have contributed to these shifts. A close relative of our study species, the striped plateau lizard *S*. *virgatus* (Wiens et al., 2010), may be an example in which this conflict has been resolved: orange, pterin‐based badges in females, distinct from those of males (Weiss et al., 2012), signal female sexual receptivity (Weiss, 2002), and predict offspring quality (Weiss et al., 2009, 2011). This case illustrates how conflict arising from mechanisms of honest signaling can lead to a different outcome in a closely related species.

In nature, conflict between sexes is pervasive, but may not necessarily lead to sexually antagonistic coevolution (Chapman, 2006). Mechanisms of honesty underlying condition‐dependent traits may hinder sexual isolation of their expression, unless they work in conjunction with sex‐specific epigenomic or physiological factors (Adkins‐Regan, 1998; Cox et al., 2017; Kimball & Ligon, 1999). In *S*. *undulatus*, the dependence of badge color saturation on androgens as a male signal of quality may have partially released females from conflict, but not fully—androgen‐independent iridophores persist in females and associate with immune response and indicators of physiological stress. On a broader scale, effects of sexual conflict can be detrimental to this species (i.e., females carry costly, purposeless, and conspicuous traits, and males make poor mate choices), and consequently, impair fitness in relation to species free from such conflicts. Further, interspecific competition between species under, and free from, sexual conflict could influence species distributions, speciation, and extinctions (Chapman, 2006; Holland & Rice, 1998; Parker & Partridge, 1998). The rich gradient of sexual dichromatism in the *Sceloporus* phylogeny (Wiens, 1999; Wiens et al., 2010) and multiple instances of liberation from potential conflict (including the evolution of female‐exclusive ornaments, Weiss, 2006) makes this genus an excellent opportunity for further understanding the broader ecological and evolutionary implications of intralocus sexual conflict over signaling traits.

## CONFLICT OF INTEREST

The authors declare they have no conflict of interest.

## AUTHOR CONTRIBUTION

**Braulio A. Assis:** Conceptualization (lead); Data curation (equal); Formal analysis (lead); Investigation (equal); Methodology (equal); Project administration (equal); Writing‐original draft (lead); Writing‐review & editing (lead). **Julian ​D. Avery:** Data curation (equal); Formal analysis (supporting); Investigation (supporting); Methodology (equal); Writing‐original draft (supporting); Writing‐review & editing (supporting). **Catherine Tylan:** Data curation (supporting); Investigation (supporting); Methodology (supporting); Writing‐original draft (supporting); Writing‐review & editing (supporting). **Heather I. Engler:** Data curation (equal); Project administration (equal); Writing‐original draft (supporting); Writing‐review & editing (supporting). **Ryan L. Earley:** Data curation (equal); Investigation (supporting); Writing‐original draft (supporting). **Tracy Langkilde:** Conceptualization (supporting); Funding acquisition (lead); Project administration (equal); Writing‐original draft (supporting); Writing‐review & editing (supporting).

## Supporting information

Supplementary Material

## Data Availability

Data are available through https://doi.org/10.26207/fzq1‐9k80.
